# URB597 induces subtle changes to aggression in adult Lister Hooded rats

**DOI:** 10.3389/fpsyt.2022.885146

**Published:** 2022-08-12

**Authors:** William G. Warren, Ed Hale, Eleni P. Papagianni, Helen J. Cassaday, Carl W. Stevenson, Christine Stubbendorff

**Affiliations:** ^1^School of Biosciences, University of Nottingham, Sutton Bonington, Loughborough, United Kingdom; ^2^School of Psychology, University of Nottingham, University Park, Nottingham, United Kingdom; ^3^Department of Neuroscience and Brain Technologies, Istituto Italiano di Tecnologia, Genoa, Italy

**Keywords:** cannabinoid, social behavior, cognition, memory, aggression, strain, Lister Hooded rats

## Abstract

The endocannabinoid system has been implicated in both social and cognitive processing. The endocannabinoid metabolism inhibitor, URB597, dose-dependently improves non-social memory in adult Wistar and Sprague Dawley rats, whereas its effect on social interaction (SI) is affected by both rat strain and drug dose. Lister Hooded rats consistently respond differently to drug treatment in general compared with albino strains. This study sought to investigate the effects of different doses of URB597 on social and non-social memory in Lister Hooded rats, as well as analyzing the behavioral composition of the SI. Males were tested for novel object recognition (NOR), social preference (between an object and an unfamiliar rat), social novelty recognition (for a familiar vs. unfamiliar rat) and SI with an unfamiliar rat. URB597 (0.1 or 0.3 mg/kg) or vehicle was given 30 min before testing. During SI testing, total interaction time was assessed along with time spent on aggressive and explorative behaviors. Lister Hooded rats displayed expected non-social and social memory and social preference, which was not affected by URB597. During SI, URB597 did not affect total interaction time. However, the high dose increased aggression, compared to vehicle, and decreased anogenital sniffing, compared to the low dose of URB597. In summary, URB597 did not affect NOR, social preference or social recognition memory but did have subtle behavioral effects during SI in Lister hooded rats. Based on our findings we argue for the importance of considering strain as well as the detailed composition of behavior when investigating drug effects on social behavior.

## Introduction

Adaptive social interaction requires the correct interpretation of social cues and subsequent adjustment of behavior to fit the situation. Both social processing and management of social behavior are impaired in psychiatric disorders, strongly impacting the ability to function in a social context (1–3). Given the prevalence of social impairment in mental illness, it is important to understand how current and novel treatments affect social cognition and behavior across patient populations. Current drug treatments for psychiatric diseases are not always effective and can induce side effects in a considerable number of patients, even after showing efficacy in preclinical trials (4, 5). The disparity in drug efficacy between preclinical and clinical studies demonstrates the importance of modeling a more diverse population in preclinical studies (6). Pharmacological treatments are commonly tested in albino rat strains (Wistar or Sprague-Dawley). However, drugs often produce different behavioral effects when tested on pigmented strains such as Long Evans and Lister hooded (LH) rats (7–11). This is possibly caused by neurobiological differences between rat strains (8–10).

The endocannabinoid (eCB) system has received increased attention as a potential target for treating a range of psychiatric disorders (12–16). The eCB system is comprised of two receptors [cannabinoid receptor type 1 and 2 (CB1R and CB2R)] and their main endogenous ligands [anandamide (AEA) and 2-arachidonoylglycerol (2-AG)], along with their respective metabolic enzymes [fatty acid amide hydrolase (FAAH) and monoacylglycerol lipase (MAGL)] (17). CB1R is preferentially expressed in the central nervous system, whilst CB2R is found more abundantly in the periphery. 2-AG has a higher affinity for CB1R and CB2R than AEA, whilst AEA shows greater affinity for transient receptor potential vanilloid 1 (TRPV1); (18). One mode of eCB transmission is *via* retrograde signaling, where 2-AG mediated activation of pre-synaptic CB1Rs leads to the inhibition of neurotransmitter release (19). AEA and 2-AG production is activity dependent and cannabinoid reuptake blockade and subsequent increase of AEA and 2-AG levels in the brain suppress glutamate release and regulate excitatory synaptic input (20). Alternatively, post-synaptic activation of TRPV1 and CB1R can increase downstream neural activity; this is largely driven by AEA (21). FAAH inhibits post-synaptic signaling by metabolizing AEA into arachidonic acid and ethanolamine, whilst MAGL metabolizes 2-AG into arachidonic acid and glycerol in the pre-synaptic terminal (22, 23). The FAAH inhibitor URB597 selectively inhibits the metabolism of AEA within 30 minutes of injection, thereby increasing its tone and availability to act on CB1R (24–26).

The eCB system modulates social behavior, cognition and memory (27–29). SI leads to increased brain AEA concentration in Wistar rats and treatment with URB597 before SI further increases brain AEA levels (30). URB597 improves non-social memory in albino rat strains (26, 31, 32), whereas it either increases or decreases social interaction in Wistar rats but does not affect social interaction in SD rats (30, 33). The evidence for these opposing behavioral outcomes of URB597 treatment suggests that social behavior may be particularly sensitive to manipulation by CB1R signaling. However, the effects of URB597 on memory and social behavior have not been tested in pigmented rat strains, such as LH. When examining drug effects on social behavior, it is important to consider changes not only to the sum but also to the specific components of social interaction. While the CB1R agonist WIN 55,212-2 reduced overall social interaction in Wistar rats, this reduction was driven by a decrease in “following” and “anogenital sniffing” of the conspecific (34). Drug-induced changes to the expression of aggression is particularly interesting when examining the impact of eCB transmission on social behavior. Violent aggression (self-directed or toward others) can be a major obstacle for treatment of psychiatric patients (35–37). Most studies examining the effects of eCBs on aggression have found cannabinoids to ameliorate expressed aggression, but some reported increased aggression in response to elevated brain AEA, with a possible link between individual differences in the level of trait aggression and drug effects (38–40). However, the effects of URB597 on overall distribution of social behavioral components and aggression remain to be elucidated. Here we characterize, for the first time, the effect of URB597 on non-social and social novelty recognition as well as its effect on social interaction and aggression in male LH rats.

## Methods

### Animals

**Figure 1:**
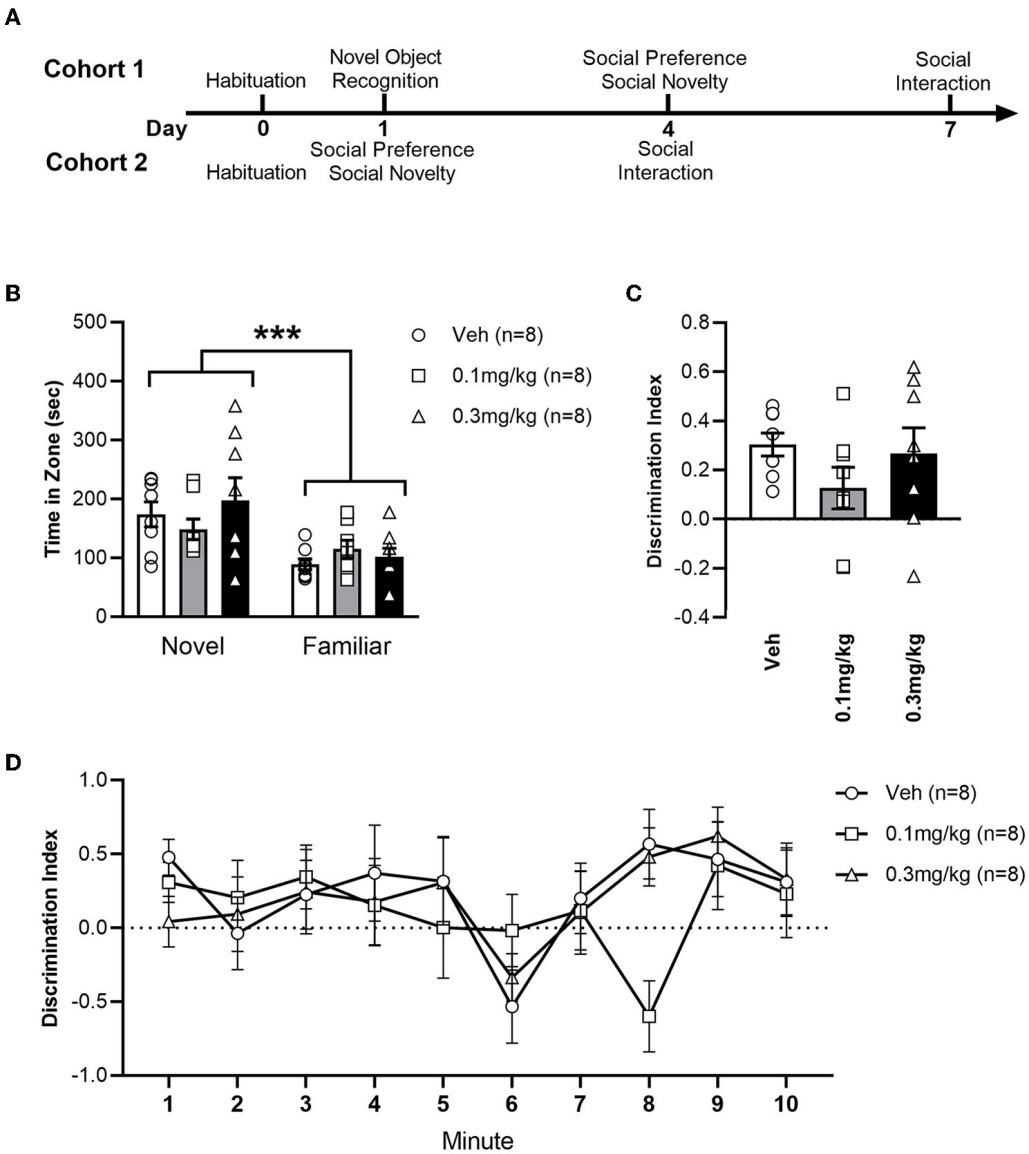
**(A)** Timeline of experiments in cohorts 1 and 2. **(B)** Average time (+/- SEM) spent exploring the novel or familiar object zone in LH rats treated with vehicle (white bar), 0.1 mg/kg URB597 (gray bar) and 0.3 mg/kg URB597 (black bar), with individual values displayed as circles, squares and triangles, respectively. **(C)** Discrimination index [(time spent exploring novel object – time spent exploring familiar object)/time spent exploring either object] for the total duration of the Novel Object Recognition test. **(D)** Discrimination index per minute. All treatment groups preferred the novel to the familiar object, while NOR was not affected by URB597. Error bars represent +/- SEM. ***p < 0.001.

The study used 48 male Lister Hooded rats (Charles River UK), weighing between 250–300 g upon arrival. Individually ventilated cages (maintained at a constant temperature of 23 ± 0.5 °C) each housed 4 rats, with a light/dark cycle of 12 h:12 h (lights on at 7AM). Food and water were available *ad libitum* whilst husbandry adhered to the principles of laboratory animal care. Each animal was tested at approximately the same time each day. Procedures used in the experiments had ethical approval from the institution's ethics committee and adhered to the UK Animals (Scientific Procedures) Act 1986 (Home Office Project License 30/3230). The animals were tested as two temporally separate cohorts of 24 animals (*n* = 8 for each treatment group in each cohort). The timeline and details of the tests in each cohort is depicted in Figure 1A.

### Drugs

URB597 (0.1 and 0.3 mg/kg; Sigma-Aldrich, UK) was solubilized in 5% polyethylene glycol (Fluka Chemicals, Switzerland), 5% Tween 80 (Sigma-Aldrich) and 90% saline; doses and protocols were based on previous findings (31–33, 41). Rats received either one of the URB597 doses or vehicle per experiment. All injections were administered intraperitoneally (1 ml/kg) and were given 30 min before testing.

### Apparatus and materials

All experiments took place in an open field arena (L: 100cm, W: 100cm) under low lighting conditions, above which a camera was positioned to record activity. Wire mesh cages were used to contain conspecifics in the social novelty test (SNT). All videos were recorded using EthoVision software (Noldus, Netherlands).

### Habituation

Day 0 – Rats were habituated to the open-field for 10 min. The time spent in the corners of the open field was recorded and the rats' preference for the corners that would contain stimuli in the following tests (see below) was calculated. This information was later used to counterbalance the placement of novel objects/conspecifics in the novel object recognition (NOR) and SNT experiments.

### Novel object recognition

Day 1 – The first phase was prepared by placing two identical objects (tin cans) in opposite corners of the open field. Test rats were individually placed in a corner of the open field, equidistant from the tin cans and allowed to explore both objects. After 10 min the rats were returned to a holding cage for 2 min while one of the tin cans was replaced with a glass jar (novel object), and an identical tin can replaced the one previously used (familiar object). In the second phase, the test rats were re-introduced to the arena for another 10 min before being returned to their home cage. Objects and the test arena were cleaned with 40% ethanol between trials. EthoVision was used to assess time spent within the two 25x25 cm corner zones containing an object.

### Social novelty test

**Figure 2:**
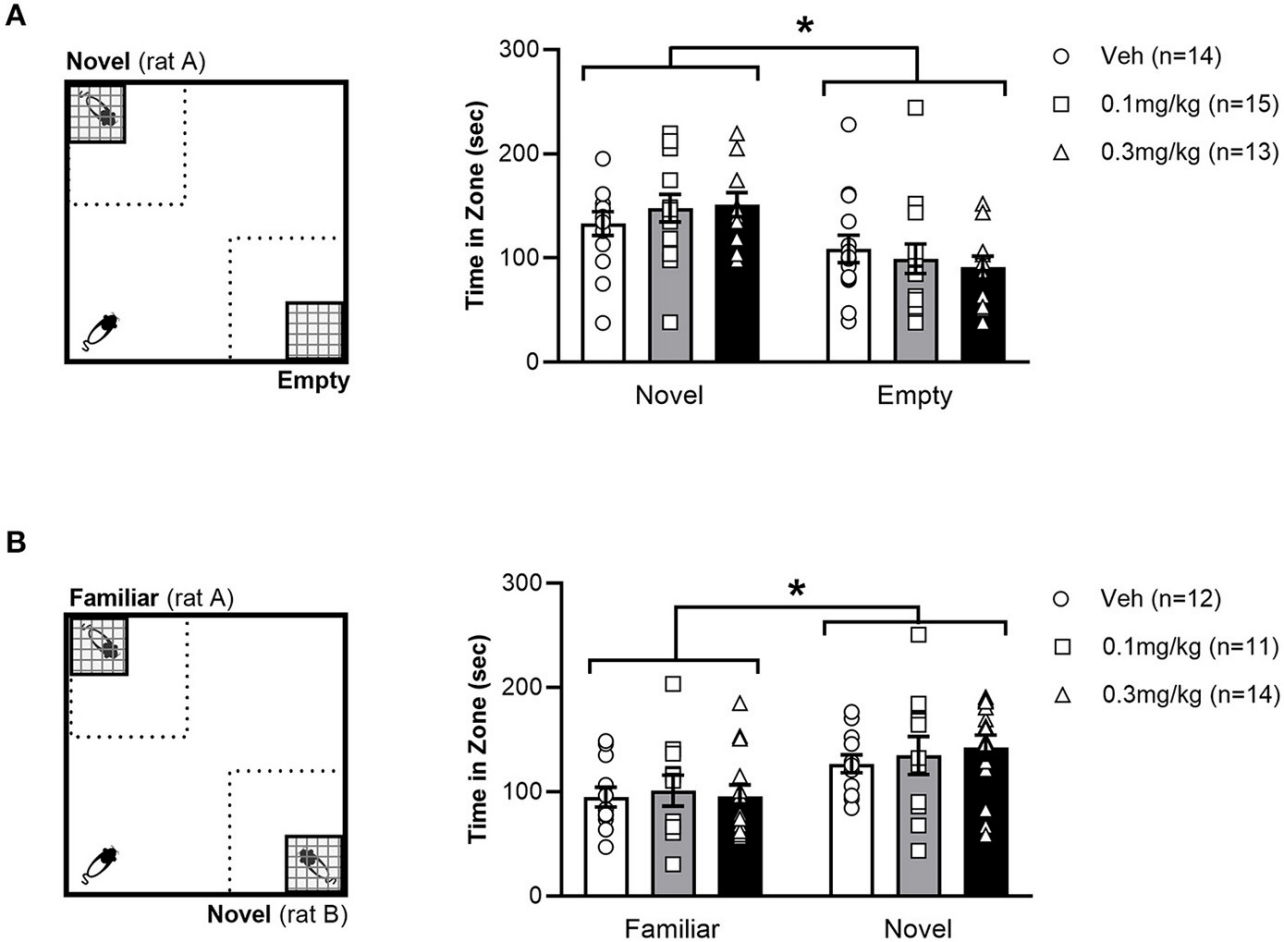
Left: Experimental set up of Social Preference test **(A)** and Social Novelty Test (SNT) **(B)**. The dotted square represents the 40x40cm interaction zones. Right: Average time (+/- SEM) spent exploring the stimulus zones during the social preference test **(A)** and SNT **(B)** in LH rats treated with vehicle (white bar), 0.1 mg/kg URB597 (gray bar) and 0.3 mg/kg URB597 (black bar), with individual values displayed as circles, squares and triangles, respectively. **(A)**. All treatment groups preferred the social stimulus (novel rat) to the non-social stimulus (empty cage), while social preference was not affected by URB597 treatment. **(B)**. All treatment groups spent more time exploring a novel unfamiliar, compared with a familiar conspecific, while social novelty preference was not affected by URB597 treatment. Error bars represent +/- SEM. *p < 0.05.

Day 1 or 4 – The SNT was adapted from Seillier and Giuffrida (42) and composed of two separate consecutive phases, depicted in Figures 2A,B. The first phase was a social preference test, where two wire mesh cages were placed in opposite corners of the arena. One cage was empty, whilst the other held an unfamiliar, weight-matched male conspecific. Test rats were placed in the corner of the arena, equidistant from both cages. The test rat then explored the open field for 10 min, after which they were removed to a holding cage. The time spent in the vicinity of the cages was recorded with EthoVision software (Noldus, Netherlands).

The second phase of the experiment tested the rat's social memory in a manner akin to the NOR. Immediately after the social preference test, a second unfamiliar weight-matched male conspecific i.e., (novel rat) was placed in the previously empty cage and then the cage was returned to its previous position. The stimulus rat used in the social preference test phase remained in its mesh cage in the same position and was now considered a familiar rat. Test rats then explored for another 10 min period before being placed back in their home cage. EthoVision calculated the time that test rats spent in the 40x40 cm interaction zone in the corners containing the cages.

### Social interaction

Day 4 or 7 – Two weight-matched unfamiliar rats received the same drug treatment and were simultaneously placed in opposite corners of an open-field for 10 min. The rats were then free to interact during this time and behavior was scored manually by an observer who was blind to the animals' treatment. Total SI was defined as time engaged in any of the following behaviors: sniffing conspecific; following; crawling over/under; and aggression. To select behavioral components for further analysis, all social behaviors were identified and scored in a subset of videos initially using behavioral categories found in the literature (43, 44). Based on this initial exploratory analysis, head/body sniffing, anogenital sniffing and aggression (boxing, pinning or biting) were selected and scored in all pairs to test for drug effects. The social behavior of each rat was scored separately and the average between interacting pairs then taken as the data output from the trial.

### Data analysis

In the NOR, data from the full 10 min session were used. However, only data from the first 5 min of the SNT were used because many of the rats moved the stimulus cages from their original corners later on during testing. Rats that moved the cages prior to the 5-min mark were removed from the analysis; 6 rats were removed from analysis of the social preference test and 11 animals were removed from analysis of both tests. Differences between groups and the zones that the rats explored were statistically assessed with a two-way analysis of variance (ANOVA). Drug treatment formed the between-subject factor, whilst zone formed the within-subject factor. For the NOR, we calculated a discrimination index defined as “time spent exploring novel object – time spent familiar object)/time spent exploring either object” (45) and analyzed discrimination index time (minute) as a repeated measure. For the SI behavioral data, one vehicle treated rat pair and one rat pair treated with 0.3 mg/kg URB597 were removed as statistical outliers based on the Grubbs test (α = 0.05) and all data from these rat pairs was omitted from the analysis. Differences between groups were assessed using one-way ANOVA, with treatment as the between-subject factor. Multiple comparisons tests were conducted using Tukey's multiple comparisons test. All data are expressed as mean ± SEM. *P* values < 0.05 were deemed significant. All videos and data analysis are available at https://osf.io/zsm8f/.

## Results

### URB597 did not affect memory in the novel object recognition test

To assess the impact of URB597 on non-social cognition, we tested our LH rats in the NOR paradigm (Figure 1). All treatment groups spent a greater amount of time investigating the novel object zone than the familiar object zone [F_(1, 42)_ = 16.66; *p* = 0.0002], demonstrating non-social novelty recognition and memory in LH rats, which is in accord with previous findings (46, 47). NOR was not affected by URB597 in our LH rats, as indicated by the lack of significant main effect of treatment [F_(2, 42)_ = 0.4736; *p* = 0.6260] or zone x treatment interaction [F_(2, 42)_ = 1.226; *p* = 0.3037]. To further examine the effect of treatment we calculated a discrimination index (Figures 1C,D). We found no effect of URB597 on NOR; neither when calculated for the total 10 min duration of the test [F_(2, 21)_ = 1.293; *p* = 0.2955], nor when examining discrimination during each minute of the test as demonstrated by the lack of main treatment effect [F_(2, 21)_; *p* = 0.6046] or minute x treatment interaction [F_(18, 189)_ = 1.044); *p* = 0.4124].

### URB597 did not affect social preference or social memory in a social novelty task

The effect of URB597 on preference for social over non-social stimuli was tested by allowing the rats to explore an empty cage (non-social stimulus) and a cage containing an unfamiliar conspecific (social stimulus) in opposite corners of an open field (Figure 2A). All treatment groups spent a greater amount of time in the zone with social stimuli than in the zone with the non-social stimuli [F_(1, 39)_ = 9.553; *p* =.0037]. However, we found no effect of URB597, as indicated by the lack of significant main effect of treatment [F_(2, 39)_ = 0.3668; *p* = 0.6953] or a zone x treatment interaction [F_(2, 39)_ = 0.5362; *p* = 0.5892], suggesting that URB597 does not alter social preference in LH rats. We then used the SNT to examine if the lack of drug effect in LH rats observed in the NOR test was specific to non-social memory (Figure 2B). Similar to the observation in the NOR test, all treatment groups spent a greater amount of time in the zone with the novel conspecific than the (now familiar) conspecific used in the social preference phase [F_(1, 34)_ = 7.235; *p* = 0.0110] but social novelty preference was not affected by URB597, as indicated by the lack of significant main effect of treatment [F_(2, 34)_ = 1.636; *p* = 0.2096] or zone x treatment interaction [F_(2, 34)_ = 0.1195; *p* = 0.8878]. These results indicate that novelty preference was consistently unaffected by URB597 in LH rats, regardless of the social or non-social nature of the novel stimuli.

### URB597 did not affect total social interaction but caused a small increase in aggressive behavior

**Figure 3:**
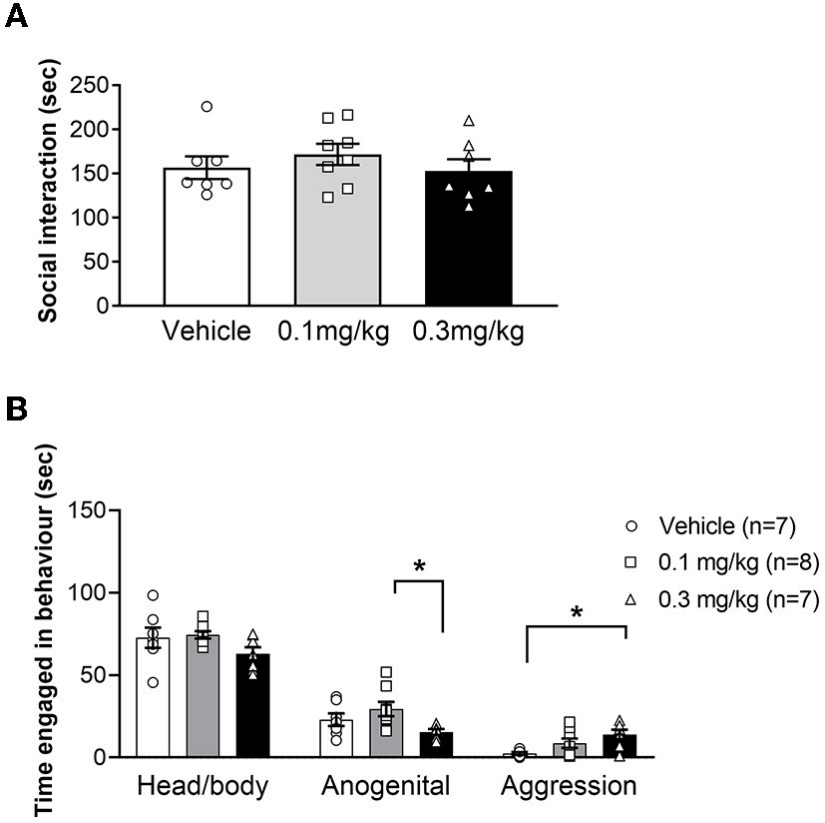
Average time (+/- SEM) spent engaged in social interaction **(A)** or specific social behaviors **(B)** in LH rats treated with vehicle (white bar), 0.1 mg/kg URB597 (gray bar) and 0.3 mg/kg URB597 (black bar), with individual values displayed as circles, squares and triangles, respectively. **(A)** URB597 had no effect on total SI in LH rats. **(B)** Rats treated with 0.3 mg/kg URB597 spent less time engaged in anogenital sniffing, compared with rats treated with 0.1 mg/kg URB597 (middle), and more time engaged in aggressive behaviors, compared with vehicle treated rats (right). Error bars represent +/- SEM. *p < 0.05.

URB597 has previously been shown to either increase or decrease social interaction in Wistar but had no effect in SD rats (30, 41, 48), therefore we decided to investigate the effects of URB597 on social interaction in LH rats (Figure 3A). We found no effect of either URB597 dose on total SI in LH rats, as indicated by the lack of significant main effect of treatment [F_(2, 19)_ = 0.8445; *p* = 0.4453]. However, we noticed that specific social behavior components, namely head/body sniffing, anogenital sniffing and aggressive behavior (boxing, pinning and biting), were more expressed in some pairs than others. We therefore assessed the effect of URB597 on these individual behaviors (Figure 3B). Whereas URB597 treatment did not alter the amount of head/body sniffing [F_(2, 19)_ = 1.010; *p* = 0.3829], LH rats treated with 0.1 mg/kg of URB597 spent more time engaged in anogenital sniffing, compared with 0.3 mg/kg of URB597 [F_(2, 19)_ = 3.754; *p* =.0423; *post hoc* test *p* < 0.05]. Finally, the higher dose of URB597 increased the amount of aggression, compared to vehicle treatment [F_(2, 19)_ = 5.336; *p* = 0.0145; *post hoc* test *p* < 0.05]. Taken together, these findings show that while URB597 did not alter total SI in LH rats, this drug appear to shift SI toward more dominance- and aggression-related behaviors.

## Discussion

### URB597 does not affect recognition memory in Lister Hooded rats

While the effects of URB597 on non-social cognition and social behavior have previously been assessed in albino rat strains, we tested its efficacy in LH rats and obtained different results compared to those reported in the literature. Previous work found URB597 (0.3mg/kg) to improve NOR in male Wistar as well as Sprague-Dawley rats (31, 32), whereas NOR performance in male LH rats in the present study was not affected by either dose of URB597. Chronic treatment with the synthetic cannabinoid receptor agonist CP55,940, impairs short-term NOR in Wistar but not in LH rats (46), suggesting that LH rats may be less sensitive to manipulation of cannabinoid receptor signaling, compared with albino rat strains. However, Renard et al. (46) also found that saline-treated LH but not Wistar rats were able to recognize a novel object after a 120 min inter-trial interval. In contrast, a more recent study showed that both Wistar and LH rats performed well in the NOR test even after a 24 h inter-trial interval (49). To fully understand the role of eCB and strain interactions on behavior, more studies using both albino and pigmented strains are needed.

In the current study, LH rats showed a preference for social vs. non-social stimuli and also displayed social novelty recognition. Previous work, using a 10 min three-chamber paradigm, reported social preference but not social novelty recognition in LH males (50). We focused on the first 5 min of the exploration, when the stimuli are most novel, which may explain why we were able to detect social novelty recognition. We found no effect of URB597 on social preference or social novelty recognition. CP55,940 dose-dependently suppressed social preference in male Wistar rats (42), demonstrating that behavior in this paradigm is sensitive to cannabinoids, at least in albino rat strains. In addition, in an experiment with intravenous self-administration, both LH and Long Evans rats acquired stable WIN 55,212-2 self-administration behavior whereas SD rats did not, further suggesting inherent differences between albino and pigmented rat strains in the response to cannabinoids (51). However, since the effect of URB597 on social preference has not been examined in albino rat strains, we cannot conclude that our findings demonstrate strain-dependent differences in eCB sensitivity.

In the current study, URB597 was solubilized in vehicle and injected 30 minutes before the onset of testing. Neurochemical analysis in Wistar rats after injection of URB597 (0.1 mg/kg) solubilized in the same vehicle found that brain AEA levels were not significantly increased until 2 h after injection (52). While injection of URB597 has been shown to affect behavior after 30 min, these studies used a different vehicle (31, 32). As we did not measure brain AEA concentrations post-injection in the present study, we cannot rule out the possibility that the lack of drug effects on NOR, social preference and novelty recognition may be explained by the interval between injection and behavioral testing, particularly in the low dose group. However, drug administration did alter behavior in the SI test, suggesting that a higher dose of URB597 injected 30 min before testing can affect behavior.

### URB597 subtly increases aggression in Lister Hooded rats

We found no effect of URB597 on the total sum of social interaction in LH rats, which is in contrast to findings in albino rats. One study found URB597 to decrease SI in Wistar rats at both low and high doses (41), whereas a different study reported that 0.1 mg/kg URB597 increased total SI in Wistar but not in SD rats (33). However, Manduca et al. (48) also observed that lighting conditions significantly affected SI and reported no effect of URB597 in either strain under low lighting conditions. In the current study, all behavioral testing was conducted under low lighting conditions, which may have contributed to the lack of drug effects.

We also quantified the sum of different behavioral components expressed during the SI test and found less anogenital sniffing in pairs treated with 0.3 mg compared with 0.1 mg of URB597. As the drug-induced shift in anogenital sniffing was not significantly different from behavior in the vehicle-treated rats, interpretation of this result can only be speculative. Previous work reports that subordinate Long Evans rats decrease the amount of anogenital sniffing after direct confrontation by a dominant rat (53) and a link between anogenital sniffing behavior and position within the dominance hierarchy has also been observed in mice (54). It is possible that the shift in anogenital sniffing in our LH rats is associated with assertion of dominance between the unfamiliar male rats. As our LH rat pairs were not re-tested to allow a dominance hierarchy to be established and assessed, it is beyond the scope of this study to draw conclusions on the role of URB597 on dominance in LH rats. However, our finding suggests that drug-induced changes to dominance-related behavior is relevant to consider when testing the effects of eCB-modulating drugs.

We observed a greater amount of time spent engaging in aggressive behavior in pairs treated with URB597 (0.3 mg/kg), compared with vehicle. To our knowledge, the effect of URB597 on aggression has not been assessed in rats before. However, in high aggressive mice, URB597 infusion into ventral hippocampus reduced aggressive behavior in the SI test (38), whereas male Syrian hamsters showed no change in brain AEA levels or defensive aggression in response to URB597 when tested as intruders in the resident/intruder paradigm (55). A study in male intruder mice in the resident/intruder paradigm reported that AEA increased territorial aggression in low-aggressive mice, while lowering territorial aggression in high-aggressive mice (40). Taken together, these findings suggest that pharmacological modulation of AEA alters the expression of aggressive behavior, but that aggression may also be influenced by the testing parameters as well as individual differences in trait aggression. However, more research sampling brain AEA during different types of aggression is needed to fully elucidate the relationship between brain AEA concentration and aggressive behaviors. When interpreting observations of increased aggression, it is important to consider that aggression in itself is not maladaptive and plays a crucial role in survival. Neither is aggression necessarily violent (e.g., in the context of competition) but when aggression manifests out of context and/or out of proportion to the situation it can be considered maladaptive (56, 57). In our SI paradigm, the rats entered the arena together and, therefore, the observed increase in aggression cannot be defined as either resident-like territorial or intruder-like defensive aggression, nor can the increased aggression be defined as maladaptive (excessive) or adaptive, e.g., competitive aggression. The observed increase in aggression manifested specifically in more boxing, pinning and biting with no injuries (puncture of skin) to either rat. While such aggressive display can be considered mild, our findings do not provide detail on the adaptive or maladaptive nature of increased aggression induced by URB597 in LH rats. Like most laboratory rodent strains, LH rats are generally low-aggressive, and therefore, our findings do not provide information about how URB597 affects aggression in a high-aggressive rat strain. However, our findings suggest that changes in eCB availability can alter levels of aggression. Furthermore, our observations demonstrate the importance of investigating drug-related changes to social behavior in more detail than merely the total sum of behavior.

### Implications of strain-dependent differences: Nuisance or opportunity?

One possible conclusion from the data on behavioral differences between albino and pigmented stains is that when designing experiments, it is important to choose a strain appropriate for the planned testing regime. However, another direction would be to embrace strain-dependent differences in the design and interpretation of experiments to model population heterogeneity, which could increase the translational value of such research (6). Investigating strain differences may reveal neurobiological differences between treatment responsive and non-responsive individuals that are relevant to understanding variability in treatment response in patients. Albino rat strains show inherent variations in brain metabolites and responses to drug treatments (8, 9, 33). Furthermore, albino and pigmented rat strains display differences in baseline behaviors as well as drug responses, possibly linked to underlying differences in synaptic processing between albino and LH strains (7, 58, 59). These findings suggest that differences among rat strains may provide valuable information regarding behavioral and neurobiological diversity relevant to modeling variability in treatment responses among patients. Of course, if the underlying factors involved in the variation of responses to cannabinoid treatment are to be properly modeled in rodents, it would be crucial to also consider sex differences. Research in both humans and rodents suggest cannabinoids are more potent in females compared with males (60, 61). In rodent models, male and female LH rats differ in brain CB1R density and function (62) and in SD rats URB597 improves NOR only in adult male but not in adult female rats (31). Taken together, these findings suggest that testing drugs in multiple rodent strains and in both females and males may reveal crucial information to understanding the variability in responses among patients.

## Conclusions

URB597 did not alter social preference and memory, NOR or the total sum of SI in LH rats, which is in contrast to findings in Wistar and SD rats, but in accord with observations of differences in responses to eCB modulation between albino and pigmented rat strains. However, differences in the interval between treatment and behavioral testing may also have played a role in the lack of effect of URB597 reported here. In the present study, neither doses of URB597 affected the total sum of social interaction in LH rats, in contrast to findings in albino strains, but the higher dose of URB597 shifted the composition of social behavior toward increased aggression. This finding underlines the importance of understanding the nature of drug-induced alterations in the expression of aggression.

## Data availability statement

The datasets presented in this study can be found in online repositories. The names of the repository/repositories and accession number(s) can be found below: https://osf.io/zsm8f/.

## Ethics statement

The animal study was reviewed and approved by the University of Nottingham Ethics Committee in adherence to the UK Animals (Scientific Procedures) Act 1986 (Home Office Project License 30/3230).

## Author contributions

WW and CStu designed the study. WW, EH, EP, and CStu conducted the experiments. WW, CSte, and CStu analyzed the data and drafted the manuscript. WW, HC, CSte, and CStu revised and approved the final version of the manuscript. All authors contributed to the article and approved the submitted version.

## Funding

WW was supported by a Biotechnology and Biological Sciences Research Council (BBSRC) Doctoral Training Partnership [Grant Number BB/M008770/1] and the University of Nottingham. EP was supported by a BBSRC Industrial CASE PhD studentship [Grant Number BB/M008770/1], which was co-sponsored by Artelo Biosciences. CStu was supported by a research grant from the BBSRC [Grant Number BB/P001149/1]. The funders had no other role in any aspect of this paper.

## Conflict of interest

WW received funding from Artelo Biosciences, which is a biopharmaceutical company with interests in the development and commercialization of cannabinoid-based medicines. The funder was not involved in the study design, collection, analysis, interpretation of data, the writing of this article or the decision to submit it for publication.

## Publisher's note

All claims expressed in this article are solely those of the authors and do not necessarily represent those of their affiliated organizations, or those of the publisher, the editors and the reviewers. Any product that may be evaluated in this article, or claim that may be made by its manufacturer, is not guaranteed or endorsed by the publisher.

## Abbreviations

2-AG: 2- arachidonoylglycerol
AEA: Anandamide
CB1R: Cannabinoid receptor type 1
CB2R: Cannabinoid receptor type 2
eCB: Endocannabinoid
FAAH: Fatty acid amide hydrolase
LH: Lister Hooded
NOR: Novel object recognition
SD: Sprague-Dawley
SI: Social interaction
SNT: Social novelty test
TRPV1: Transient receptor potential vanilloid 1.
